# Regressing SARS-CoV-2 Sewage Measurements Onto COVID-19 Burden in the Population: A Proof-of-Concept for Quantitative Environmental Surveillance

**DOI:** 10.3389/fpubh.2021.561710

**Published:** 2022-01-03

**Authors:** Itay Bar-Or, Karin Yaniv, Marilou Shagan, Eden Ozer, Merav Weil, Victoria Indenbaum, Michal Elul, Oran Erster, Ella Mendelson, Batya Mannasse, Rachel Shirazi, Esti Kramarsky-Winter, Oded Nir, Hala Abu-Ali, Zeev Ronen, Ehud Rinott, Yair E. Lewis, Eran Friedler, Eden Bitkover, Yossi Paitan, Yakir Berchenko, Ariel Kushmaro

**Affiliations:** ^1^Central Virology Lab, Ministry of Health, Sheba Medical Center, Jerusalem, Israel; ^2^Avram and Stella Goldstein-Goren, Department of Biotechnology Engineering, Ben-Gurion University of the Negev, Beer Sheva, Israel; ^3^Department of Life Sciences, Ben-Gurion University of the Negev, Beer Sheva, Israel; ^4^School of Public Health, Sackler Faculty of Medicine, Tel-Aviv University, Tel Aviv, Israel; ^5^Zuckerberg Institute for Water Research (ZIWR), Blaustein Institutes for Desert Research, Ben-Gurion University of the Negev, Sde Boker, Israel; ^6^Maccabi Healthcare Services, Tel-Aviv, Israel; ^7^Faculty of Medicine, Technion–Israel Institute of Technology, Haifa, Israel; ^8^Faculty of Civil and Environmental Engineering, Technion–Israel Institute of Technology, Haifa, Israel; ^9^Department of Chemical Engineering, Technion–Israel Institute of Technology, Haifa, Israel; ^10^Clinical Microbiology Laboratory, Meir Medical Center, Kfar Saba, Israel; ^11^Department of Industrial Engineering and Management, Ben-Gurion University of the Negev, Beer Sheva, Israel; ^12^The Ilse Katz Center for Meso and Nanoscale Science and Technology, Ben-Gurion University of the Negev, Beer Sheva, Israel

**Keywords:** surveillance, wastewater based epidemiology, sewage, corona, COVID-19, SARS-CoV-2, virus concentration

## Abstract

Severe acute respiratory syndrome coronavirus 2 (SARS-CoV-2) is an RNA virus, a member of the coronavirus family of respiratory viruses that includes severe acute respiratory syndrome coronavirus 1 (SARS-CoV-1) and the Middle East respiratory syndrome (MERS). It has had an acute and dramatic impact on health care systems, economies, and societies of affected countries during the past 8 months. Widespread testing and tracing efforts are being employed in many countries in attempts to contain and mitigate this pandemic. Recent data has indicated that fecal shedding of SARS-CoV-2 is common and that the virus RNA can be detected in wastewater. This indicates that wastewater monitoring may provide a potentially efficient tool for the epidemiological surveillance of SARS-CoV-2 infection in large populations at relevant scales. In particular, this provides important means of (i) estimating the extent of outbreaks and their spatial distributions, based primarily on in-sewer measurements, (ii) managing the early-warning system quantitatively and efficiently, and (iii) verifying disease elimination. Here we report different virus concentration methods using polyethylene glycol (PEG), alum, or filtration techniques as well as different RNA extraction methodologies, providing important insights regarding the detection of SARS-CoV-2 RNA in sewage. Virus RNA particles were detected in wastewater in several geographic locations in Israel. In addition, a correlation of virus RNA concentration to morbidity was detected in Bnei-Barak city during April 2020. This study presents a proof of concept for the use of direct raw sewage-associated virus data, during the pandemic in the country as a potential epidemiological tool.

## Introduction

Waterborne pathogens, including viruses, bacteria, and protozoa can be shed into the urban water cycle *via* sewers (1, 2), urban runoff, agricultural runoff, and wastewater discharges (3, 4). Treated wastewater is often used for agriculture and industrial and may cause biological and environmental concerns (5, 6). Fecal indicator bacteria are often used as a marker for the microbial quality of the treated effluents but obviously, they have limitations in representing viral pollution.

Coronavirus severe acute respiratory syndrome coronavirus 2 (SARS-CoV-2) is a novel RNA virus belonging to a group of viruses that includes amongst others severe acute respiratory syndrome (SARS) and Middle East respiratory syndrome (MERS). SARS-CoV-2 is one of more than 37 coronaviruses in the *Coronaviridae* family, within the order *Nidovirales*, and it is currently causing a major pandemic with millions of infected people globally. It causes COVID-19, a disease that has a daunting effect on health care systems, economies, and societies of affected countries. As a member of the *Coronaviridae*, which includes viruses known to cause respiratory and/or intestinal infections, SARS-CoV-2 spreads primarily *via* microdroplets, reflecting its survivorship in humid environments (7). Recent reports have detailed SARS-CoV-2 shedding in the human stool (8–10). Interestingly it has been demonstrated that a similar coronavirus, SARS-CoV-1 can survive in sewage for 14 days at 4°C, and 2 days at 20°C, and its RNA can be detected for 8 days, even though the virus was inactive (11, 12). Recently, the SARS-CoV-2 virus was detected in wastewater in treatment facilities (9, 13–16). Despite this, we are still lacking sufficient studies regarding the fate of SARS-CoV-2 throughout the different stages of wastewater collection and treatment processes (17) and its ultimate fate at the end of the treatment process.

The presence and prevalence of SARS-CoV-2 in wastewater provide a valuable epidemiological data source (18). Wastewater-based epidemiology (WBE) is a new discipline concerned with mining chemical and biological information from municipal wastewater. WBE has been applied to populations around the globe to measure chemicals consumption and exposure patterns (19). It has been proven to be useful for preclinical identification (i.e., before the population exhibited symptoms) of the Aichi virus for monitoring antibiotic resistance on a global scale (18), for quantitative polio surveillance (20), and also provides fecal indicators (2, 21). In a previous study (20), valuable epidemiological information regarding polio was obtained by analyzing two unique data sets collected during a “natural experiment” provided by the 2013 polio outbreak in Israel. In that study, wastewater data from different locations and records of supplemental immunization with the live vaccine were correlated. The parametric characterization of the linear dose-dependent relationship between the number of poliovirus shedders and the amount of poliovirus in sewage yielded a powerful tool for quantitative environmental surveillance (20). Here we report a study aimed at developing similar tools for SARS-CoV-2 in wastewater. These results will enable spatial-based monitoring of future outbreaks and could be used to confirm virus elimination in the wastewater treatment terrain, and to validate the need for additional containment efforts.

## Materials and Methods

### Sampling

Samples were taken from wastewater treatment plants in different locations in Israel (see **Table 3**). For the examination of virus concentration methods, samples of raw sewage from Dan-Panaroma hotel, Tel Aviv (termed here TLV1-10), and from Zfat (termed here Z1-5) were collected. The sampling equipment was sanitized and properly sterilized (for example, cooler, sampling bottles, and biohazard bags). In addition, for sampling different geographic locations (Supplementary Figure S1), we used automatic samplers at targeted hot-spot areas. Around 200 ml were collected every 30 min for 24 h at each site. Samples from the automatic sampler (6–10 L) were transported immediately to the lab where they were poured into 2L clean plastic bottles. The samples were divided between the Environmental Biotechnology lab (BGU) and the Central virology lab (CVL). Fresh 1 ml raw sewage was placed directly into the lysis buffer for RNA extraction. The rest of the sample was stored at −20°C or −80°C for the virus concentration and RNA extraction stages.

### Sample Extraction Methods

Sewage is composed mostly of water and may include detergents and organic matter, which may have a great impact on inhibition when using molecular methods. Therefore, to decrease the effect of inhibitors, several extraction kits (**Table 2**) were used to ascertain a preferable kit for SARS-CoV-2 RNA extraction. Four raw sewage samples from areas with numerous COVID-19 populations were spiked with MS2 and human coronavirus CO43 and were analyzed by different kits to evaluate which kit works best with wastewater. The spiked sewage samples were stirred for 30 min, divided into aliquots, and kept at −80 degrees. Several RNA extraction kits were tested to optimize the removal of impurities from the raw sewage samples. The kits used were; AccuPrep® Viral RNA Extraction Kit (Bioneer); NucleoSpin RNA, NucleoSpin RNA Stool and NucleoSpin RNA Virus (Macherey Nagel, Gemany), Quick-DNA/RNA Viral MagBead (Zymo Research, CA, United States), and Monarch® Total RNA Miniprep Kit (New England BioLabs, MA, United States).

### Sample Concentration and Analysis

A few concentration methods were used to achieve improved Ct (Threshold detection cycle in qPCR) values. Viral particles from ~0.25 to 1 liter of sewage samples were first centrifuged to remove sediment and large particles. Virus precipitation of the supernatant was performed using different methodologies: Polyethylene glycol (PEG) 8,000 or 6,000, alum (20 mg/l, unless mentioned otherwise), skim milk solution (0.01% w/v in sewage), or ultrafiltration. The sewage mixtures were mixed at 4°C with 100-rpm agitation for 12 h, followed by centrifugation at 4,500–14,000 g for 45 min at 4°C to pellet the virus particles. The virus particles were then resuspended in phosphate-buffered saline (PBS). The aqueous phase (containing virus particles) was collected and filtered through a 0.22 μm filter. The TLV2 sample was mixed with glycine buffer (0.05 M glycine, 3% beef extract) at 1:4 volume ratios before the precipitation. The mixture was incubated at 4°C with 100-rpm agitation for 2 h, to detach virions bound to organic material. To continue in optimizing precipitation methods, different precipitant weight percentages were tested using different salt (NaCl) concentrations (Table 1). In some cases, the initial centrifugation step was replaced with a rough filtration step using paper filters (TLV5, TLV6, and TLV7). For the TLV8 sample, 50 mL of raw sewage sample passed through 0.22 μm vacuum filter (Sterilflip, Milipore Express PLUS Membrane) and 1 mL of PBS backwash from the filter was collected. TLV9 and TLV10 passed through folded filter paper or were centrifuged at 4°C, 3000g for 5 min respectively. The samples were then concentrated to 1 mL using Centrifugal Filter Unit with 10 KDa (Milipore). Samples were stored at −20/−80°C until further analysis. Viral RNA was extracted from the samples using a viral RNA extraction kit (RNeasy mini kit- QIAGEN or EasyMAG -bioMerieux, France) and then stored at −80°C.

**Table 1:** Severe acute respiratory syndrome coronavirus 2 (SARS-CoV-2) virus concentration methods from sewage.

**Sample**	**Method principle**	**Concentration factor**	**Target gene**	Ct before concentration^*^	Ct After concentration^*^	**Lab**
TLV1	Concentrated using the polyethylene glycol (PEG) precipitation method. The sample was gently mixed with PEG 6000 and 0.92 g of NaCl for 12 h at 4°C	100	N	35.8	33.1	BGU
TLV2	Glycin buffer prior to concentration in order to detach virions bound to organic material. The sample filtered through 0.22 um and virus precipitation continued using PEG 8000 (80 g/L) and NaCl (17.5 g/L)	100	N	35.8	ND	BGU
TLV3	PEG precipitation (10% PEG 8000 (w/v) and 0.3 M NaCl) after pH adjustment (pH = 7.2). The pellet was extracted with chloroform	100	N	35.8	ND	BGU
TLV4	Concentrated using Aluminium Sulfate. The sample was gently mixed with 20 mg/mL Alum for 12 h at 4°C	100	N	35.8	33.6	BGU
TLV5	Folded filter paper and then concentration using Aluminium Sulfate. The sample was gently mixed with 10 mg/mL Alum for 12 h at 4°C	50	N	35.01	ND	BGU
TLV6	Folded filter paper and then concentration using Aluminium Sulfate. The sample was gently mixed with 50 mg/mL Alum for 12 h at 4°C	50	N	35.01	36.41	BGU
TLV7	Folded filter paper and then concentration using Aluminium Sulfate. The sample was gently mixed with 100 mg/mL Alum for 12 h at 4°C	50	N	35.01	38.43	BGU
TLV8	The sample was filtered 0.22 um and then backed flash	50	N	34.32	33.21	BGU
TLV9	Folded filter paper followed by Centricon (10 kDa cutoff)	10	N	34.32	36.58	BGU
TLV10	Short centrifugation at 4oC, 3000g for 5 min followed by Centricon (10 kDa cutoff)	10	N	34.32	36.85	BGU
Z1	Concentrated using the polyethylene glycol (PEG) precipitation method. The sample was gently mixed with PEG 6000 and 0.92 g of NaCl for 12 h at 4°C. Then centrifugation 4500g for 45 min and discarding the supernatant. The pellet was collected by PBS + Tween (0.05%) buffer without the chloroform step.	50	E	33.37	33.29	CVL
Z2	Concentrated using the polyethylene glycol (PEG) precipitation method. The sample was gently mixed with PEG 6000 and 0.92 g of NaCl for 12 h at 4°C. Then centrifugation 4500g for 45 min and discarding the supernatant. The pellet was collected by PBS + Tween (0.05%) buffer without the chloroform step.	50	E	ND	ND	CVL
Z3	Preliminary centrifugation (2500g) before precipitation method. Concentrated using the polyethylene glycol (PEG) precipitation method. The sample was gently mixed with PEG 6000 and 0.92 g of NaCl for 12 h at 4°C. Then centrifugation 4500g for 45 min and discarding the supernatant. The pellet was collected by PBS + Tween (0.05%) buffer without the chloroform step.	50	E	ND	35.76	CVL
Z4	Concentrated using skim milk precipitation method. The sample was gently mixed with 0.01% (w/v) skim milk for 12 h at 4°C. Then centrifugation 4500g for 45 min and discarding the supernatant. The pellet was collected by PBS + Tween (0.05%) buffer without the chloroform step.	50	E	ND	ND	CVL

* *ND, not detected/undetermined (Ct > 40)*.

### Identification and Quantification of Coronavirus

In the BGU laboratory, the extracted viral RNA samples were tested using the BGI commercial kit (https://www.bgi.com/global/sars-cov-2-real-time-fluorescent-rt-pcr-kit-ivd). Quantitative PCR amplification was performed in a Step One Plus real-time PCR system (Applied Biosystems, Thermo Scientific, Massachusetts, USA). Both positive-control and negative-control assays were performed for quality control. In CVL, positive controls for the SARS-2 E and N genes were generated, by synthesizing RNA fragments corresponding to the amplified regions in the qPCR test. Serial dilutions of these synthetic RNA fragments were used to generate the standard curves of plasmid log copy number vs. Ct value (see Supplementary Material). The thermal profile of the reverse transcription PCR (RT PCR) test was based on the protocol published by Corman et al. and was adjusted for the use with SensiFast reaction mix (Bioline, https://www.bioline.com/us/), thereby shortening the time of the reaction, while maintaining its sensitivity. The test was successfully performed with both ABI7500 Bio-Rad CFX-96 instruments. The analytical sensitivity was tested and was found to be comparable to that described by Corman et al. The analytical limit of detection (LOD) of the E gene target was <10 target copies per reaction for the E gene.

## Results and Discussion

In this study, we examined virus concentration methods from sewage samples (Table 1). These methods were validated using sewage samples collected from the Dan Panorama hotel in Tel Aviv, Israel. This hotel functioned as a COVID-19 isolation facility in March and April 2020. In addition, sewage from Zfat that was spiked with inactivated SARS CoV-2 was used to test the efficiency of the concentration methods on viral retrieval from sewage. Results showed that none of the concentration methods were able to achieve significant improvement in the RT PCR Ct values. Hence, in general, little improvement in virus concentration was observed using these methods. Despite this, TLV1 and TLV4 reached positive values with the RT PCR Ct of 33.1 and 33.6 for PEG and Alum, respectively (Table 1), making them the best of the different methods tested. Surprisingly, filtrations steps did not seem to improve virus concentration. This has led us to hypothesize that perhaps part of the viral particles and/or viral RNA fragments are attached to sewage-organic particles resulting in loss of viral RNA in the concentration process. Indeed, Ye et al. (22) showed that PEG and Ultracentrifugation poorly recover enveloped viruses from sewage. For the most part, recent publications reporting SARS-CoV-2 detection in municipal wastewater, report no comparison of Ct values before and after concentration procedures, making it difficult to determine if the concentration methods were able to recover the virus efficiently (17, 23–25).

Since raw sewage samples contain high levels of impurities that later affect and inhibit molecular enzymatic reactions, it is important to clean these inhibitors before the reverse transcription and qPCR. Specialized environmental RNA extraction kits usually contain sample cleanup steps. In Table 2, we present 14 different RNA extraction kits that were examined using 200 μL raw sewage samples. ZYMO magnetic beads kit showed poor ability to extract RNA from raw sewage. It is probably due to the presence of impurities that block the beads from binding to RNA molecules. The EasyMag kit, on the other hand, showed good performance in extracting the RNA. In that case, 1 mL of raw sewage was extracted following quick centrifugation to remove large particles. A second RNA extraction kit that showed good results was the Macherey Nagel stool kit, where there is a column clean-up step after the lysis step followed by a different column that binds the RNA.

**Table 2:** Detection of SARS-CoV-2 using different RNA extraction kits.

**RNA extraction kit**	N gene SARS-CoV-2 Sample 1 (Ct)^*^	E gene SARS-CoV-2 Sample 2 (Ct)^*^
Bioneer (hylab)	36.7	-
New England Biolabs	35.24	-
Macherey Nagel Viral RNA	35.41	-
Macherey Nagel stool RNA	34.34	-
Qiagene Rneasy mimi kit	35.12	-
ZYMO magnetic beads	ND	-
Eazymag	-	33.6
QIAGEN microbiom	-	ND
zymo fecal/soil microbe	-	37.4
QIAGEN PowerViral	-	37.6
promega	-	35.7
Zymo Direct-zol RNA Miniprep	-	34.4
EPICENTER	-	35.77
SEEGENE (STRALET)	-	37.45

* *ND, not detected/undetermined (Ct > 40)*.

**Figure 1:**
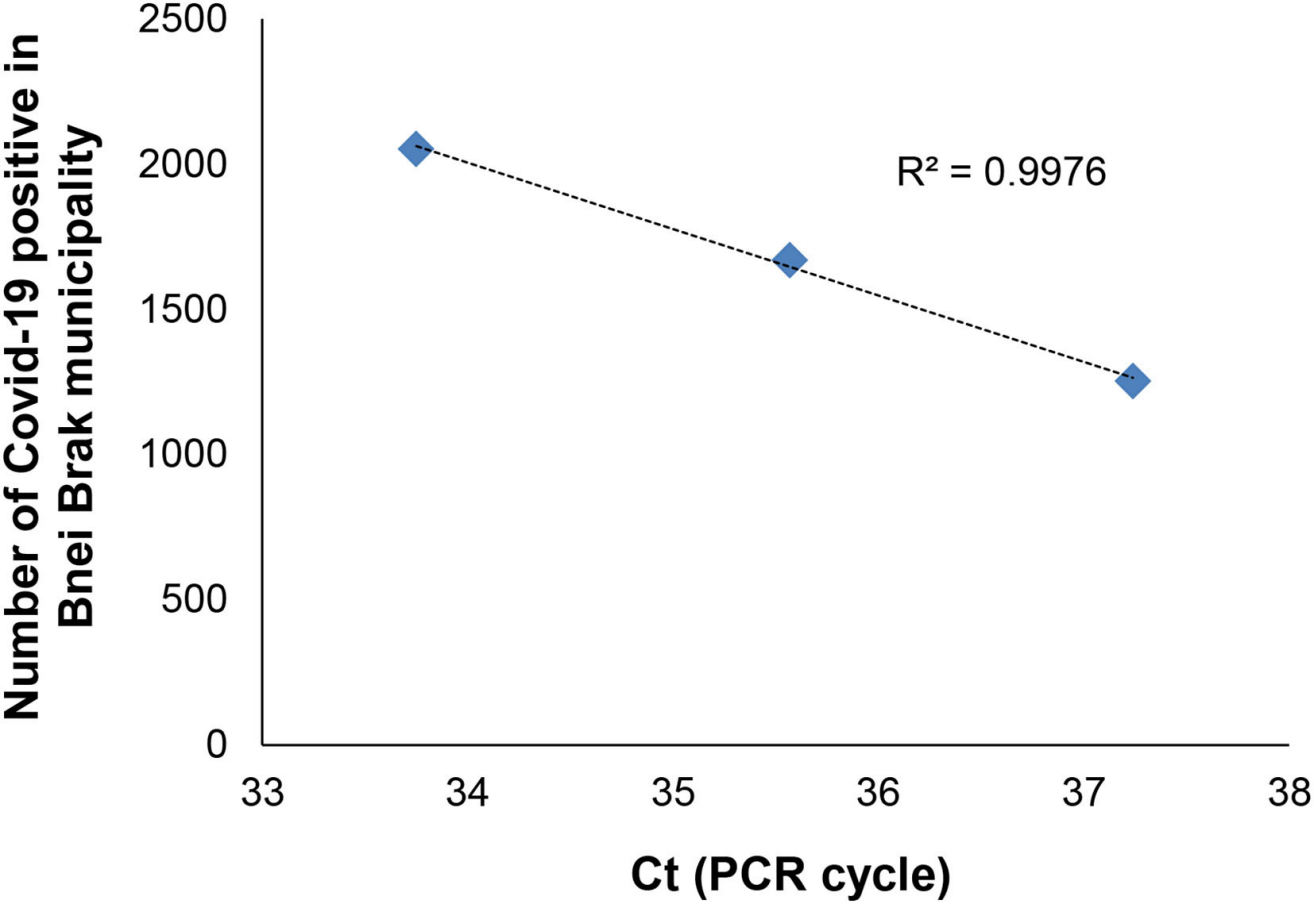
SARS-CoV-2 Ct in raw sewage (RT-PCR) vs. the number of positive diagnosed Covid-19 in Bnei Brak city during April 2020.

In this study, we further established a proof-of-concept for the ability to detect SARS-CoV-2 RNA from raw sewage samples from different geographical locations (Table 3). We found traces of the virus in sewage originating from the Sorek wastewater treatment plant (Ct 32.9) as well as from Bnei Brak sewage sampling points (Ct 33-37). The concentration of the virus RNA (as Ct) from the Bnei Brak sewage correlated with the general number of COVID-19 positive individuals in the city during April 2020 (see Figure 1). Furthermore, the change observed in the Jerusalem sampling points from the end of March to the 21st of April demonstrates the dynamics of the COVID-19 outbreak in the community (Table 3). Interestingly the Beer Sheva, and Haifa, samples were negative (>Ct 40) for SARS-CoV-2, possibly related to the low proportion of infected people in these cities (Table 3). For active COVID-19 cases in sampling sites from which wastewater was examined, see also Supplementary Figure S2. Additional research groups engaging in COVID-19 monitoring in urban wastewater, detected some correlation between total COVID-19 morbidity and Ct values in wastewater (17, 23–25). In general, the higher the reported COVID-19 morbidity, the lower the detected Ct value (higher count of template RNA) in the area's wastewater. Despite this, it is not possible at this point to determine the real nature behind this correlation, as there are too many degrees of freedom in the modeled system. Limited clinical surveillance in terms of testing the entire population, inaccurate diagnosis, limited data accessibility, as well as proper characterizations of the wastewater are all significant variables to consider when trying to decipher the correlation between clinical results and wastewater diagnostics. Therefore, it is imperative to further characterize such a correlation.

**Table 3:** Proof-of-concept for detecting SARS-CoV-2 from raw sewage in different geographic localities in Israel.

**Name of place**	Main characteristic^**^	**Date of sampling**	**SARS-CoV-2 (Ct)**	**Adenovirus (Ct)**	**MS2 (Ct)**	**# of positive for COVID-19**
Haifa	WWTP	10-03-2020	ND^*^	30.85	ND	
Shafdan	WWTP	10-03-2020	ND	30.48	ND	
Rahat	WWTP	25-03-2020	ND	31.14	ND	
Arara	WWTP	25-03-2020	ND	30.17	ND	
Beer Sheva	WWTP	25-03-2020	ND	31.13	33	
Ayalon	WWTP	25-03-2020	ND	30.59	ND	
Zfat	WWTP	26-03-2020	ND	33.33	33.53	
El Hamra	WWTP	26-03-2020	ND	33.82	33.29	
Haifa	WWTP	26-03-2020	ND	31.32	ND	
Haifa	WWTP	26-03-2020	ND	ND	ND	
Sheba Hospital	HC (SN)	30-03-2020	33.22	36.9	34.43	
Kidron (Jeruselam)	WWTP	30-03-2020	ND	31.22	29.42	
Sorek (Jeruselam)	WWTP	30-03-2020	38.5	32.9	27.58	
Og (Jeruselam)	WWTP	30-03-2020	ND	31.13	ND	
Haifa	WWTP	30-03-2020	ND	32.79	ND	
Dan Panorama Hotel	IF (SN)	03-04-2020	38.03	ND	31.37	
Shmoel Ha Roffea	HC (SN)	03-04-2020	ND	ND	ND	
Bnei Brak	SN	03-04-2020	37.24	32.14	32.46	1,253
Bnei brak	SN	03-04-2020	35.57	32.88	36.1	1,669
Haifa	WWTP	05-04-2020	ND	33.73	ND	
Dan Panorama Hotel	IF (SN)	13-04-2020	35.51	ND	ND	
Bnei Brak	SN	13-04-2020	33.75	36.59	32.68	2,052
Nir David	SN	15-04-2020	ND	36.72	ND	
Nir Etzion	IF (SN)	16-04-2020	32.76	47.3	ND	
Sorek (Jeruselam)	WWTP	21-04-2020	34.66	32.99	ND	
Og (Jeruselam)	WWTP	21-04-2020	36.95	32.38	24.42	

* *ND, not detected/undetermined (Ct > 40)*.

** *HC, Hospital treating SARS-CoV-2 patients; IF, Isolation facility; SN, Sewer network; WWTP, wastewater treatment plant*.

In conclusion, we present a preliminary study demonstrating a proof-of-concept for the detection of SARS-CoV-2 RNA in raw sewage. We present two methods for viral isolation from wastewater by concentration with PEG and/or alum. Importantly our study presents a linear “dose-dependent” curve as a tool for viral surveillance in environmental samples with high viral loads in sewage reflecting the infection in the area assessed. However, we urge the readers to be cautious in their use of Figure 1 as a basis for their own data since (i) our previous study (20) indicated how different localities should be compared by considering daily sewage production as a measure of the local population size, and by the fact that (ii) our work is still preliminary and ongoing, therefore further data is still warranted. Understanding the ecological dynamics of SARS-CoV-2 in human waste could lead to efficient monitoring and surveillance of this virus as well as to provide an additional application for environmental surveillance. In the future, this study may also provide tools for sewage monitoring as an early warning alarm for SARS-CoV-2 outbreaks in the population.

## Data Availability Statement

The raw data supporting the conclusions of this article will be made available by the authors, without undue reservation.

## Author Contributions

IB-O and KY executed all experiments, analyzed and wrote this manuscript. MS, EO, MW, VI, ME, and OE participate in experiment execution. IB-O, KY, OE, YP, ON, ZR, EM, HA-A, BM, and RS contributed to method development. EK-W performed language editing. EB, ER, YL, EF, and YB were in charge of sampling design, execution and data analysis. AK and YB wrote this manuscript and took part in experimental design.

## Funding

We would like to thank funding from the Ben Gurion University, the Corona Challenge Covid-19 (https://in.bgu.ac.il/en/coronachallenge/Pages/default.aspx) and funding from the Israeli ministry of Health.

## Conflict of Interest

The authors declare that the research was conducted in the absence of any commercial or financial relationships that could be construed as a potential conflict of interest.

## Publisher's Note

All claims expressed in this article are solely those of the authors and do not necessarily represent those of their affiliated organizations, or those of the publisher, the editors and the reviewers. Any product that may be evaluated in this article, or claim that may be made by its manufacturer, is not guaranteed or endorsed by the publisher.
