# Supplementary material for: Regressing SARS-CoV-2 Sewage Measurements Onto COVID-19 Burden in the Population: A Proof-of-Concept for Quantitative Environmental Surveillance
Source: Front Public Health. 2022 Jan 3;9:561710. doi: 10.3389/fpubh.2021.561710 (PMC8762221; doi:10.3389/fpubh.2021.561710)
Supplement: Supplementary file 1 [file Image_1.pdf]

Supplementary Material

Regressing SARS-CoV-2 Sewage Measurements Onto COVID-19 Burden in the Population: A Proof-of-Concept for Quantitative Environmental Surveillance

Itay Bar-Or [1†], Karin Yaniv [2†], Marilou Shagan[2] , Eden Ozer [3] , Merav Weil[1] , Victoria Indenbaum[1] , Michal Elul[1] , Oran Erster 1 , Ella Mendelson[1,4], Batya Mannasse[1] , Rachel Shirazi[1] , Esti Kramarsky-Winter [2] , Oded Nir [5] , Hala Abu-Ali[5] , Zeev Ronen[5] , Ehud Rinott[6] , Yair E. Lewis[7] , Eran Friedler[12] , Eden Bitkover[8] , Yossi Paitan[9] , Yakir Berchenko[10 *] and Ariel Kushmaro[2,11]

1 Central Virology Lab, Ministry of Health, Sheba Medical Center, Jerusalem, Israel.

2 Avram and Stella Goldstein-Goren, Department of Biotechnology Engineering, Ben-Gurion University of the Negev, Beer Sheva, Israel.

3 Department of Life Sciences, Ben-Gurion University of the Negev, Beer Sheva, Israel.

4 School of Public Health, Sackler Faculty of Medicine, Tel-Aviv University, Tel Aviv, Israel.

5 Zuckerberg Institute for Water Research (ZIWR), Blaustein Institutes for Desert Research, Ben-Gurion University of the Negev, Sde Boker, Israel.

6 Faculty of Health Science, Ben-Gurion University of the Negev, Beer Sheva, Israel.

7 Faculty of Medicine, Technion–Israel Institute of Technology, Haifa, Israel.

8 Department of Chemical Engineering, Technion–Israel Institute of Technology, Haifa, Israel.

9 Clinical Microbiology Laboratory, Meir Medical Center, Kfar Saba, Israel.

10 Department of Industrial Engineering and Management, Ben-Gurion University of the Negev, Beer Sheva, Israel.

11 The Ilse Katz Center for Meso and Nanoscale Science and Technology, Ben-Gurion University of the Negev, Beer Sheva, Israel.

12 Faculty of Civil and Environmental Engineering, Technion–Israel Institute of Technology, Haifa, Israel.

Supplementary Material

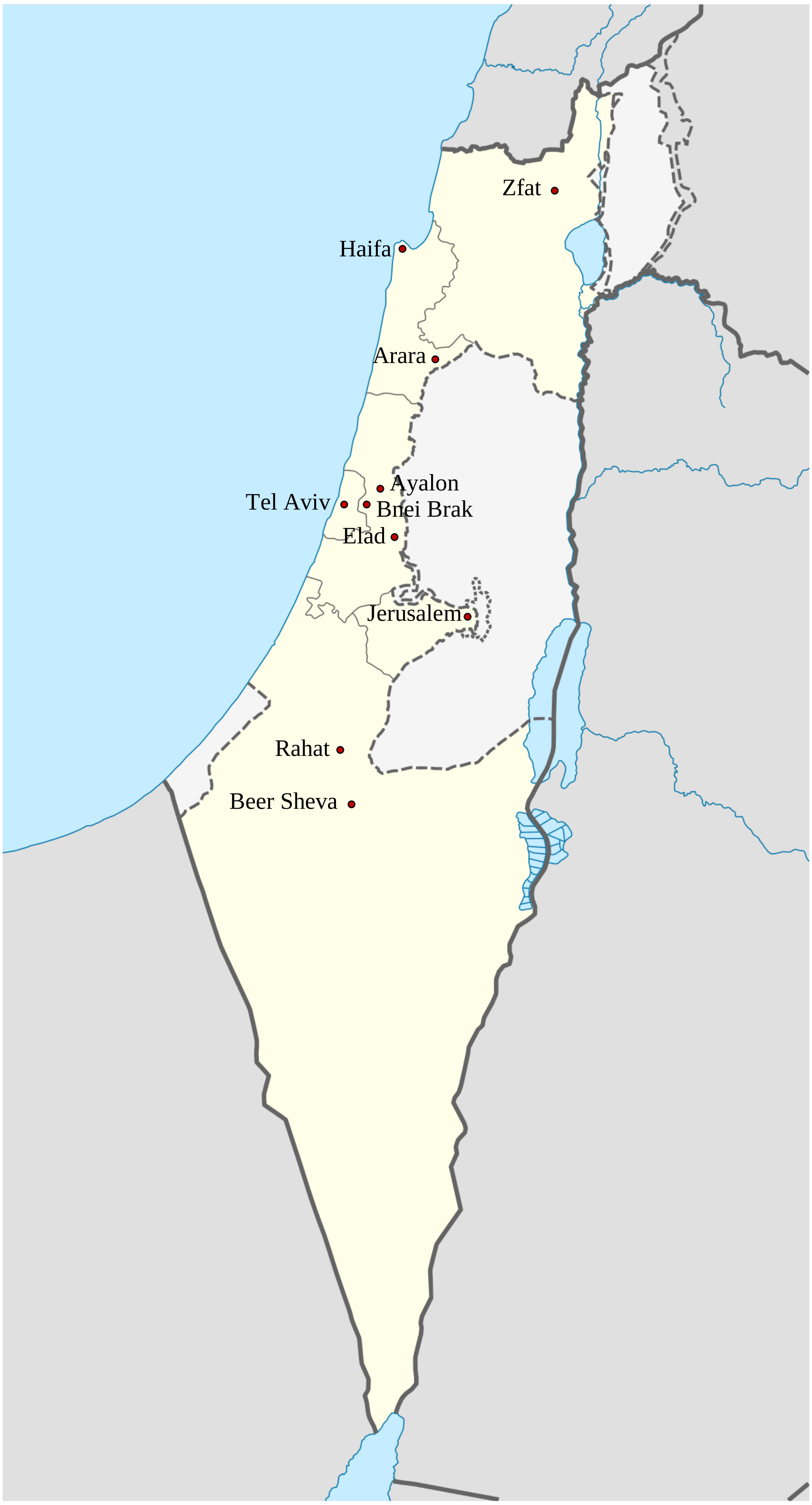

Figure S-1. Main geographic locations from which wastewater were sampled
