# Supplementary figures and images for: Regressing SARS-CoV-2 Sewage Measurements Onto COVID-19 Burden in the Population: A Proof-of-Concept for Quantitative Environmental Surveillance
Source: Front Public Health. 2022 Jan 3;9:561710. doi: 10.3389/fpubh.2021.561710 (PMC8762221; doi:10.3389/fpubh.2021.561710)

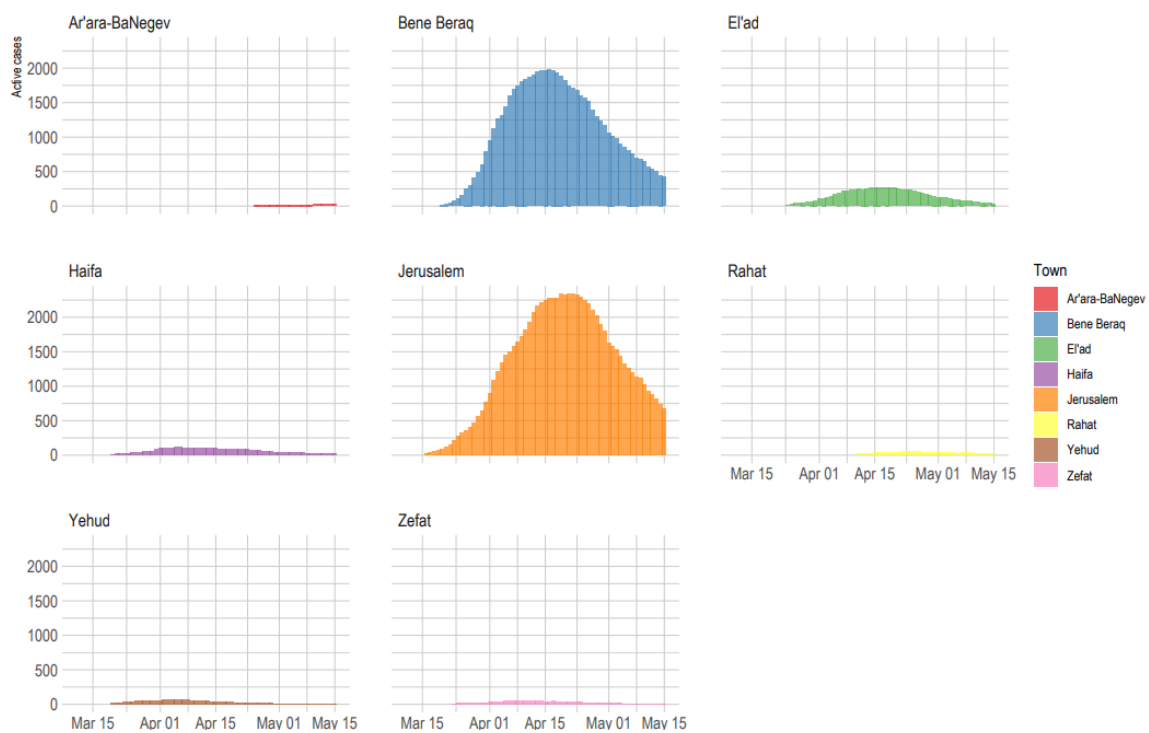

Figure S-2. Active COVID-19 cases in sampling sites from which wastewater was examined

Supplement: Supplementary file 2 [file Image_2.pdf]
